# Introducing visual participatory methods to develop local knowledge on HIV in rural South Africa

**DOI:** 10.1136/bmjgh-2016-000231

**Published:** 2017-09-28

**Authors:** Chloe Brooks, Lucia D’Ambruoso, Karolina Kazimierczak, Sizzy Ngobeni, Rhian Twine, Stephen Tollman, Kathleen Kahn, Peter Byass

**Affiliations:** 1 Department for International Development, London, UK; 2 Centre for Global Development and Institute of Applied Health Sciences, University of Aberdeen, Scotland, UK; 3 Umeå Centre for Global Health Research, Umeå University, Umeå, Sweden; 4 MRC/Wits Rural Public Health and Health Transitions Research Unit (Agincourt), School of Public Health, Faculty of Health Sciences, University of the Witwatersrand, Johannesburg, South Africa; 5 Business School, University of Aberdeen, Scotland, UK; 6 INDEPTH: An International Network for the Demographic Evaluation of Populations and Their Health, Accra, Ghana

**Keywords:** HIV/AIDS, participatory action research, health systems, health systems research, rural, South Africa.

## Abstract

**Introduction:**

South Africa is a country faced with complex health and social inequalities, in which HIV/AIDS has had devastating impacts. The study aimed to gain insights into the perspectives of rural communities on HIV-related mortality.

**Methods:**

A participatory action research (PAR) process, inclusive of a visual participatory method (Photovoice), was initiated to elicit and organise local knowledge and to identify priorities for action in a rural subdistrict underpinned by the Agincourt Health and Socio-Demographic Surveillance System (HDSS). We convened three village-based discussion groups, presented HDSS data on HIV-related mortality, elicited subjective perspectives on HIV/AIDS, systematised these into collective accounts and identified priorities for action. Framework analysis was performed on narrative and visual data, and practice theory was used to interpret the findings.

**Findings:**

A range of social and health systems factors were identified as causes and contributors of HIV mortality. These included alcohol use/abuse, gender inequalities, stigma around disclosure of HIV status, problems with informal care, poor sanitation, harmful traditional practices, delays in treatment, problems with medications and problematic staff–patient relationships. To address these issues, developing youth facilities in communities, improving employment opportunities, timely treatment and extending community outreach for health education and health promotion were identified.

**Discussion:**

Addressing social practices of blame, stigma and mistrust around HIV-related mortality may be a useful focus for policy and planning. Research that engages communities and authorities to coproduce evidence can capture these practices, improve communication and build trust.

**Conclusion:**

Actions to reduce HIV should go beyond individual agency and structural forces to focus on how social practices embody these elements. Initiating PAR inclusive of visual methods can build shared understandings of disease burdens in social and health systems contexts. This can develop shared accountability and improve staff–patient relationships, which, over time, may address the issues identified, here related to stigma and blame.

**Key questions**

**What is already known about this topic?**

- The HIV/AIDS epidemic in South Africa has had devastating impacts on health, society and economy. Despite progress in treatment and life expectancy, the epidemic continues to evolve and present new challenges.
- A range of health system factors have been demonstrated to influence HIV mortality in rural South Africa. There is, however, comparatively less focus on social factors, and how health system and social factors affect experiences of living with HIV.
- Health systems require evidence for policy and programming that goes beyond burden of disease. HIV/AIDS-related mortality is widely acknowledged to be determined by social and health systems issues. Social analysis with an equity focus can help to understand and inform policy and other interventions.

**What are the new findings?**

- We initiated a Participatory Action Research (PAR) process, inclusive of a qualitative visual element (Photovoice), to elicit local knowledge on HIV/AIDS-related mortality and identify priorities for action. PAR transforms the roles of those participating towards more active roles as coresearchers and agents of change.
- We used social theory consistent with models of health systems as core social institutions to further develop explanations of the data and analysis.
- In this study, evidence was developed and actions appraised, but it was not possible to take action within the time and resources available. Through sustained engagement with local communities and health systems stakeholders, however, support has been gained to scale up the process into a multisectoral reflection and action cycle.

**Key questions**

**Recommendations for policy**

- Policy interventions that focus on routine and habitual social processes and practices that shape health and well-being in disadvantaged and vulnerable groups may provide a route through which to address HIV-related mortality.
- The initialised PAR process, visual evidence and theory-informed analysis significantly contribute to advancing understandings of HIV-related mortality. When embedded in health systems, the process may have further potential to generate robust evidence with practical relevance and inform remedial action.

## Introduction

Within and between countries, the social determinants of health inequalities are widely acknowledged to account for significant proportions of avoidable mortality and morbidity.^1^ As such, health issues need to be set in their broader social contexts to be fully understood and effectively acted on. The research presented in this article was developed in South Africa, a country with deeply entrenched social and health inequalities.^2 3^

Legacies of colonialism and apartheid are clearly seen in differentials of opportunity, health and wealth in South Africa today.^4^ Under apartheid, non-whites had political representation removed and citizenship withdrawn.^2^ Public services were racially segregated, with services for non-whites greatly inadequate in comparison with those provided for the white population.^2^ Despite the end of apartheid in 1994, pervasive racial discrimination continues.^4^ Economic inequalities are also entrenched: the wealthiest 10% receive 58% of the annual national income, whereas the poorest 70% receive 17%.^4 5^ Forty-five per cent of the population live on or below the poverty line, with high unemployment in rural areas and among young people.^6 7^

Rural areas, in particular, are affected by this legacy. Fewer whites lived in rural areas, and so healthcare in these areas was historically not a priority for policy makers. Today, rural–urban divides are observed in HIV prevalence and healthcare.^8^ For example, HIV prevalence is considerably lower at 18% in Western Cape, a more urbanised province, than in KwaZulu-Natal where the prevalence is 40%.^9^

Despite entrenched social and health inequalities, the postapartheid policy context in South Africa is progressive and inclusive. Under the Bill of Rights in South Africa’s Constitution, everyone has the right to access healthcare services,^10^ and in 2011, a White Paper for National Health Insurance was published in a bold commitment to Universal Health Coverage.^11^ Despite this, there is chronic underinvestment in public services, human resource crises, corruption, poor stewardship and deteriorating infrastructure, resulting in deep disconnects between policy and implementation.^3^

In this context, the health system faces a quadruple burden of disease comprising infectious and non-communicable diseases (NCDs), mortality owing to violence and maternal and child causes.^6 12–14^ HIV/AIDs is a major challenge. Although South Africa accounts for 0.7% of the global population, it shoulders 17% of the global burden.^6^ South Africa has more people living with HIV (PLHIV), 7 million people, than any other country.^14^ The national prevalence is 17%, but in some regions, including the study setting, prevalence rates have been observed in excess of 40%.^9 15^

HIV rates are socially patterned, and gender disparities are particularly pronounced. Incidence among  women aged 15–24 years is four times higher than that of men,^12^ with women in this age group occupying a low position in society, often with limited power in relationships.^16^ Gender inequalities also converge with economic and racial inequalities, placing significant strains on the health system.^17–19^ For individuals, families and the country, the high cost of treating HIV feeds the cycle of poverty entrenching socially patterned burdens of mortality and morbidity.^20^ Stigma compounds economic inequality via social exclusion, negatively affecting health outcomes through reduced mental and social well-being, and as barriers to testing and treatment.^21^

Progress in containing the epidemic accelerated after the national Anti-Retroviral Therapy (ART) programme was initiated in 2004.^22^ It is now the largest ART programme in the world, providing treatment for over 2.5 million people.^23^ Estimates suggest that 42% of adults with HIV are currently receiving ART in South Africa.^14^ This figure is expected to rise following the initiation of Universal Test and Treat (UTT) in September 2016. UTT is based on WHO guidelines under which lifelong ART is initiated within 14 days of a positive test result, irrespective of CD4 count.^24^ South Africa is one of the first countries to implement the policy.^25^

The success of ART has contributed to a considerable increase in life expectancy for PLHIV, from 52 to 63 years from 2003 to 2015.^26^ New priorities have emerged over this period, however, with increasing prevalence of NCDs^27 28^ and complex comorbidities in poor and black populations.^20^ This applies to other vulnerable populations: as elderly populations increase, efforts to ensure that their voice and needs are represented in service organisation are also important.^29^

Prevention is widely accepted to be the most cost-effective solution to HIV/AIDS.^30^ It is also recognised that effective prevention must address underlying social and cultural factors.^31^ Despite this, however, preventative approaches comprised just 11% of the HIV budget in 2011–2016.^32^ Prevention is challenging due to persisting stigma, the fear of which propels the epidemic via secrecy and denial.^21^ Convenience, confidentially and credibility have been posited as important to people to improve the acceptability of testing and treatment and, consequently, health outcomes.^33^

A notable exception is the prevention of mother-to-child transmission programme, with South Africa on course to eliminate vertical transmission,^34^ and with considerable reductions in under-five mortality observed recently.^35^ These gains are informing current directions, with the UTT programme arguably acting both curatively and preventatively.

Evidence has played an important role in HIV/AIDS in South Africa. At the beginning of the epidemic, the causes and scale were not acknowledged and actively disputed by the authorities.^36^ In this context, consistent and reliable mortality data became instrumental in encouraging evidence-based policy responses.^37^ Accurate data on HIV-related mortality, however, remain irregular and disparate across the country. This acts as both a cause and a consequence of poor overall developmental progress more broadly.^37–39^

Evidence comes in different forms. In public health, qualitative evidence is often neglected in favour of findings from quantitative research.^40–42^ This is due, in part, to qualitative research being viewed as less rigorous, even when quantitative criteria of validity and reliability are inappropriately applied. As set out above, HIV/AIDS is clearly patterned by social and economic disadvantage. It follows that social analyses, with an explicit equity focus, can help to understand the issues and from this basis effectively inform policy and other forms of intervention.

Participatory action research (PAR) is a relativist approach concerned with equity in terms of social exclusion from health systems.^43^ PAR encourages the active involvement of research participants and, through sustained engagement, it aims to encourage participants to develop and adopt roles as agents of change.^44^ PAR seeks to democratise the process between researcher and participants and empower participants through this process. The use of PAR in health systems research has been emphasised recently as an approach to create new knowledge and provoke action.^45^

Insights into health systems functioning are also important. According to a people-centred perceptive, health systems are core social institutions, whose structure and functions reflect and reinforce community values and norms of how people interact with and navigate the system.^46–48^ Choices, and trust, in health systems enable individuals to exercise control, acting as agents in influencing health and life situations. According to this viewpoint, the effectiveness of the health system correlates with the quality of human relationships involved.

### Aims and objectives

The aim of the research was to gain insights into the perspectives of rural South African communities on HIV-related mortality. The objectives were to initiate a process to elicit and organise local knowledge and to identify priorities for service organisation and delivery. The approach acknowledged the value of local knowledge to provide insights that complement other forms of health information, here with reference to data from demographic surveillance.

## Methods

The study was conducted at the Agincourt Health and SocioDemographic Surveillance System (HDSS), in Mpumalanga province, rural northeast South Africa. Agincourt HDSS is a longitudinal population registration system encompassing 31 villages, totalling a population of 110 000, served by seven public clinics, two public health centres and one private health centre.^49^

Since 1992, Agincourt HDSS has provided continuous longitudinal surveillance data to improve understandings of health, population and social transitions. The site is located in Mpumalanga, a rural province of 4 million people in the northeast bordering Swaziland and Mozambique. Conditions in villages are comparable in the region: there is inadequate sanitation, poor infrastructure, high population density and high levels of unemployment.^49^ The burden of HIV is high and highly unequal. Prevalence in black populations is 40–50 times that of white populations, and for adolescents, risks are 8 times higher in females than males.^50^ In the site, age-standardised HIV prevalence is 26% for women and 19% for men.^51^

We initiated a PAR process in Agincourt HDSS. PAR is a distinct type of research in which people with common interests engage in observing, reflecting, acting and learning from action.^45^ In the time available (18 months, preceded by 12 months of predevelopment work), it was possible only to initiate a process to elicit local knowledge and appraise priorities for action. We, therefore, based our approach on the following description: ‘start by obtaining and insight into the communities and their conditions. This provides the information to support inclusion in the work, to systematise experience and to draw out priorities for attention’.^45^ We asserted that although we could not commit to taking action, and learning from action, it was worthwhile to initiate a process as a basis from which to gain support to move towards fuller forms of PAR.

To prioritise and maintain prior partnerships in communities, we attempted to reconnect with people involved in previous pilot work in Agincourt, entitled ‘Public Health Evaluation of Verbal Autopsy (PHEVA)’.^52^ In the earlier study, three village-based discussion groups had been convened, including women, family members, traditional healers, religious leaders, community health volunteers, community health workers and community leaders to examine and appraise verbal autopsy (VA^i^) data from Agincourt HDSS. In the earlier work, one group had exclusively female members to minimise any potential bias arising from power differentials in mixed groups.

Agincourt HDSS staff approached participants of the previous study, described the new research study called VAPAR (Verbal Autopsy and Participatory Action Research), planned activities and expected outputs. We informed participants that we would (1) examine VA data from Agincourt HDSS, further verify and amplify these data with local knowledge and identify priorities for action; (2) present the evidence and appraise the priorities for action with representatives of the provincial Department of Health; and (3) use the initialised process as a basis from which to develop an ongoing partnership with the Department of Health. Those who wished to be involved were provided with written informed consent forms and information, and a mutually convenient time was arranged for the first meeting. All 24 participants involved in the earlier work were recruited, and the three village-based discussion groups were reconvened (table 1).

**Table 1:** Composition of village-based discussion groups

Participants*	Group			Total
	A	B	C	
Women of reproductive age	1	1	2	4
Family members†	2	2	2	6
Traditional healers	1	1	2	4
Religious leaders and elders	1	2	2	4
Community health volunteers‡	1	1		2
Community/village officials‡	1	1		2
Community/village health workers‡	1	1		2
Number of participants, total	8	8	8	24
Proportion participants, female:male	75:25	50:50	100:0	75:25

*All participants recruited were 18 years or older. Although participants were categorised by more than one role in the community, one role per individual was considered for participant recruitment. We agreed roles with participants to identify what they felt to be their primary role in the community.

†Close relative: parents, grandparents, siblings, children, in-laws, nieces, nephews and cousins.

‡We acknowledged that people with working arrangements, particularly village health workers and village officials, may not be available for a series of six weekly meetings. We also acknowledged the ethical imperative of engaging participants who would otherwise be involved in earning income and/or the provision of public services. The groups were convened with careful consideration of minimising disruption to local public services.

The groups then operated independently through a series of six weekly meetings (table 2). In each group’s first meeting, we asked about the conditions to examine to encourage participant control over how topics were selected and framed. We also consulted a provincial planning directorate and considered burdens of disease as determined through Agincourt HDSS data. HIV-related mortality and under-five mortality were selected through this process. This paper focuses on HIV; the findings on under-five mortality are presented elsewhere.^53^

**Table 2:** Schedule of village-based meetings

Week	1	2	3	4	5	6
Topic/ Group	Introduction and recruitment	Under-five mortality		HIV-related mortality		
		Life histories and collective analysis	Collective analysis (cont.) and action agendas	Life histories and collective analysis	Collective analysis (cont.) and action agendas	Preliminary feedback and reflections on process
A	A, 1	A, 2	A, 3	A, 4	A, 5	B, 6
B	B, 1	B, 2	B, 3	B, 5	B, 5	B, 6
C	C, 1	C, 2	C, 3	C, 5	C, 5	C, 6
Total number of meetings						18

In subsequent meetings, HIV was discussed with reference to causes, consequences and modifiable factors. A senior qualitative facilitator from Agincourt presented HDSS mortality data on levels of, and circumstances surrounding, HIV-related deaths in Agincourt HDSS. This was followed by facilitated discussions on causes, contributory factors and priorities for action from the perspectives of the discussion groups.^54 55^ We worked through a sequence in which life histories were elicited through discussions on participants’ knowledge about recognition of conditions, modern and traditional therapies, availability and quality of services and what happens in acute situations.

We then developed collective views on the issues and on priorities for action. Shared accounts of participants’ views on HIV mortality were developed using ranking and diagramming. We recorded the main points from the discussion on a flip chart visible to all participants and summarised and checked the list for completeness when the discussion was concluded. Participants then deliberated over and organised the initial list by voting for issues of highest relevance. We held two voting rounds to capture the collective opinion of the group and used adhesive markers to encourage participation and symbolise representation.

In the following meeting, the ranked list was checked again and further organised using a problem tree diagram. The problem tree helped the group to consider, reorganise and reanalyse the causes and contributory factors of HIV mortality as proximate, intermediate and distal.^45^ In the final meeting for each group, we invited participants’ views on actions to reduce the burden of HIV-related mortality that addressed issues identified, and we deliberated over how these might be achieved locally. In the final discussion, we revisited the prior process and appraised remedial actions.

One of the discussion groups used Photovoice, a visual participatory method in which participants use photography to represent community conditions, physical environments, assets and other phenomena.^56^ Participants in the remotest, all female group (group C) were provided with digital cameras to collect visual data on relevant aspects of the local environment. For group C, we provided a general introduction to photography, an explanation of the need to secure release permissions from subjects of photographs and supplies of permission release forms.

In the weekly meetings in this village, images recorded by participants were an integral part of the discussions. In each meeting, the images were collated and projected so that participants could (1) present their images and describe their rationales for taking them as illustrations of factors influencing HIV-related mortality and (2) interrogate and discuss these images with the group, as further inputs to the deliberative process. This provided an additional means through which to elicit participants’ subjective perspectives and to systematise these into collective forms of knowledge. The approach allowed for the participants’ analyses of images to be captured in the narratives and, by extension, in the narrative analysis (see below). The images and accompanying narratives, thus, formed an important element of both deliberative and analytical processes.

Topic guides were used to structure the meetings, which were held in the local language of xiTsonga. With separate consent, the meetings were audio recorded and thereafter transcribed verbatim and translated into English. A field assistant, who supported the facilitator, translated and transcribed the audio recordings. The transcriptions were checked for accuracy and meaning against the recordings by the facilitator and assistant. In group C’s meetings, photographs taken by participants were also collated, displayed and critiqued.

A thematic analysis was conducted in parallel to, and following completion of, the data collection to complement and illustrate the participants’ analysis. An inductive/deductive framework analysis approach was adopted in this analysis.^57^ Inductive analysis allowed meaning to emerge from the data, whereas deductive analysis involved the use of codes defined a priori. This acknowledged the study design and prior knowledge, while also allowing unanticipated findings to be identified and developed.

After familiarisation with the data, codes were identified and assigned.^58^ Coding continued iteratively until no new codes emerged or data were coded. Codes were then grouped into themes and subthemes, updating the framework during the process. The visual data were triangulated with the discussion data, taking note of the narratives of group C participants presenting and explaining their photographs.^59^

Institutional Review Boards at the Department of Health and Social Development in Mpumalanga, Universities of the Witwatersrand in South Africa and of Aberdeen in the UK approved the study protocol. Written informed consent was gained from all participants, and further consent was gained for reproduction of photographs. We also reimbursed participants travel expenses and gave each participant a shopping voucher of ZAR300 (approximately US$20) at the end of the process. Participants were assured that all identifiable data would be anonymised in transcriptions and reports. Participants who used Photovoice received additional training on photography and release permissions.^60^

## Findings

An analysis of community perspectives on HIV-related mortality is presented below. The causes, contributory factors and priorities identified by participants are arranged according to two overall categories: (1) social and (2) health systems factors. We use verbatim quotes and visual images to illustrate the analysis, which is also summarised in table 3. Permissions have been secured for the reproduction of all images.

**Table 3:** Thematic framework

	Social	Health systems
Causes/contributors	Alcohol use and abuse Gender inequalities Problems with informal care and poor sanitation Harmful traditional practices Stigma and disclosure	Delays in treatment Problematic staff–patient relationships Blame towards staff Lack of confidentiality Lack of trust
Priorities for action	Youth facilities Improved employment opportunities Government intervention	Expanded community-based engagement and health promotion Timely treatment and outreach

## Social factors

### Alcohol use and abuse

Alcohol use and abuse were identified as causative by all discussion groups. Alcohol was said to contribute to the transmission of HIV via unprotected intercourse, with several references to alcohol and sexual violence. Participants also reported views that alcohol neutralises ART effectiveness and can lead to non-adherence.

*Man: ‘Rape is common and is happening … especially to those who drink alcohol’. (Group A)*

### Gender inequalities

Unequal power in relationships was identified more generally as a cause and contributor of HIV. Marked differences were observed between the perspectives of men and women on this topic. Male participants expressed views that power is equal in relationships and that decisions for safe sex are controlled by women. Although this suggests that men perceive that women are, or should be, able to make such decisions, it also could be seen as a means to avoid responsibility. As stated by a male participant,

*Man: ‘I can’t take decisions for my partner just because I am a man’. (Group A)*

Female participants, by contrast, asserted that inequality in relationships is subtle and pervasive, resulting from societal inequalities that shape behaviours, and that there is lack of autonomy to challenge these. Men were also described as higher wage earners, with greater power in relationships as a result. Power asymmetries in relationships were particularly pronounced among older men involved with younger women. A notable finding was that female participants often expressed blame towards young women, despite their lack of power and control in these situations.

*Woman: ‘Girls will keep on dying because they like money’. (Group C)*

**Figure 1:**
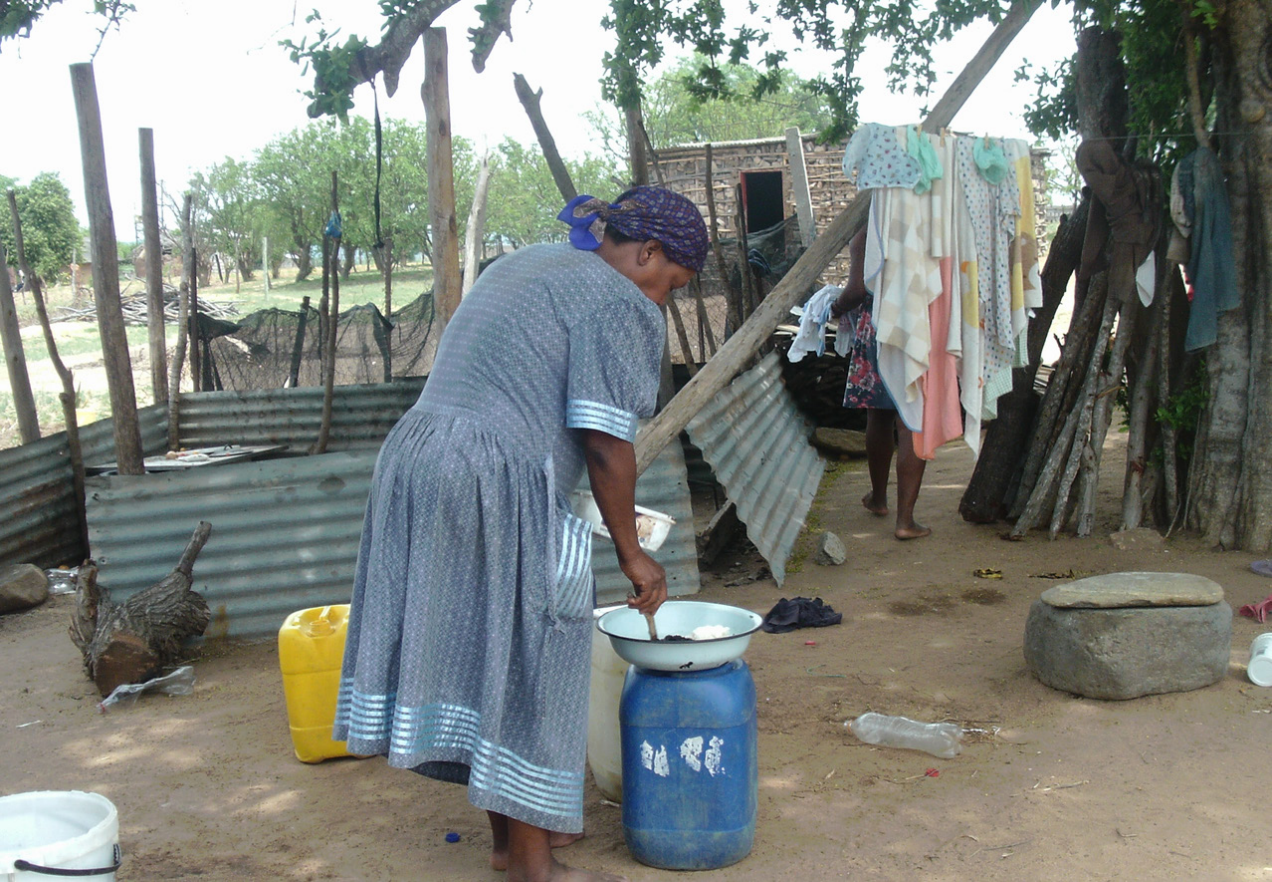
Widow living in poor conditions (Photovoice image) Woman: ‘Those women you see there…they are many, so I found them on the loan shark because of poverty… they don’t have husbands… I told them that the government has to see how we suffer… sometimes the sun sets without eating anything.’ (Group B).

Women were reported to be disproportionately affected financially after losing a partner to HIV/AIDS. Participants recounted how women in financial need use informal lenders operating illegally and charging inflated interest rates with impoverishing consequences (figure 1).^61^ Otherwise, women generally viewed men having a greater role in spreading the disease via infidelity. Men, by contrast, perceived men and women as equally to blame.

*Woman: ‘Men are the ones who go around and see different women…’ (Group C)*

*Man: ‘…people believe they are 50/50 [responsible for infidelity], she says ‘if he goes, I will also go my way and then we will meet in the morning when we come back’. (Group C)*

### Problems with informal care and poor sanitation

**Figure 2:**
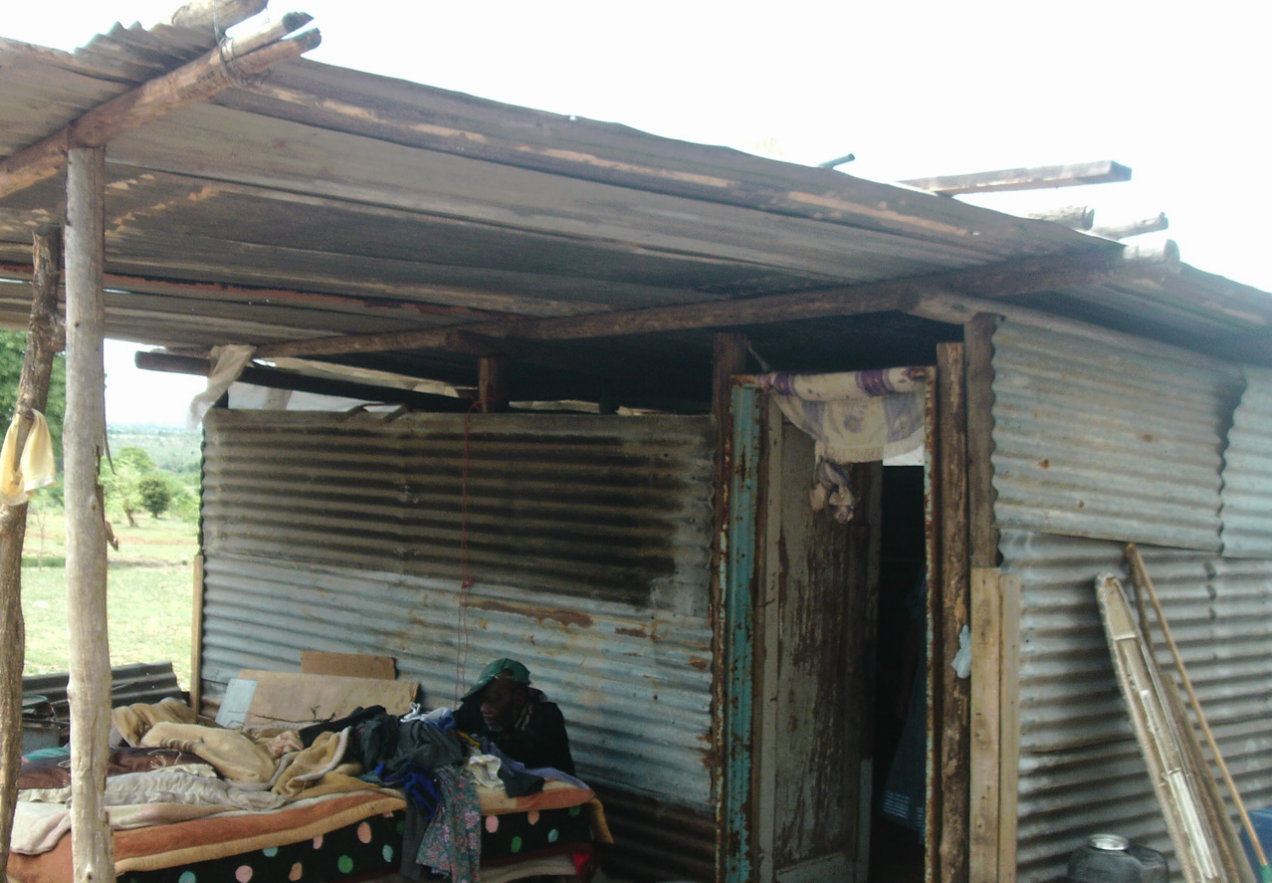
Sub-standard accommodation (Photovoice image) Woman: “These people are sick… she has… STI’s…her husband looks like he’s also taking HIV treatment meaning that is two treatments” (Group B).

Due to overall costs of care and treatment, the importance of care outside the health system and support from friends or family were highlighted. Participants noted that how HIV can push families into poverty from indirect costs (eg, transport, food, gloves and painkillers) (figure 2) and that caring for PLHIV put caregivers at risk if they cannot afford protective supplies.

**Figure 3:**
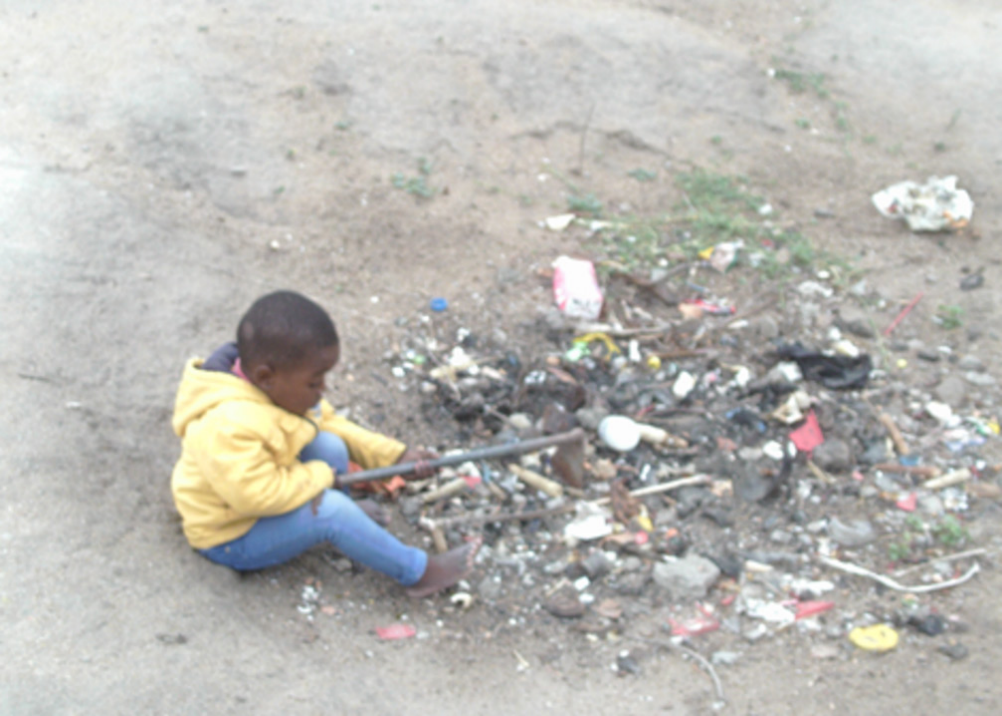
Unsafe environment (Photovoice image) Woman: “This child is sick and there is no one who is taking care of him, the mucus falls and where he lives is not clean. So it shows that he’s not safe and he could get infected with TB and also HIV.” (Group B).

**Figure 4:**
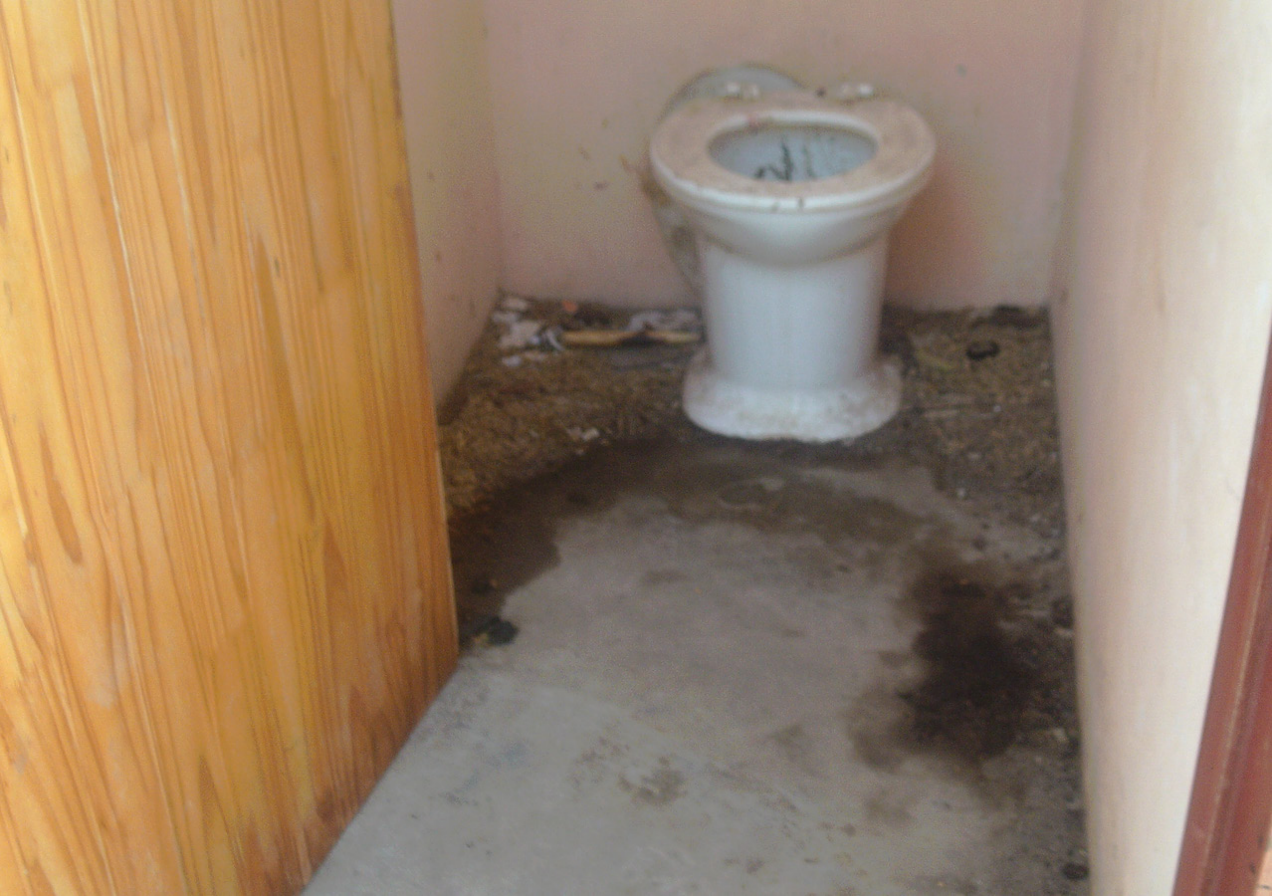
Inadequate sanitation (Photovoice image) [please include carriage return]  Woman: “Their toilets don’t have doors… they don’t wash hands, they just take their food and eat.” (Group B).

In a similar sense to the blame expressed towards young women, the parents of HIV-positive children were often portrayed as cruel, blamed for lack of care and for mother-to-child transmission (figure 3), and schools were generally viewed as not achieving child health promotion and care, including a lack of proper supervision. Inadequate sanitation in schools was a further issue identified (figure 4), and a lack of trust in government provided public services was also expressed.

*Woman 2: ‘Parents are cruel… [they say] ‘I don’t have time to nurse my child. I go around sleeping with men, the time to give the child [ART] passed because I am still in my boyfriend’s place’’. (Group C)*

### Harmful traditional practices

Some traditional practices were described as contributing to HIV transmission through the use of razors in ceremonies and practices. Preferences for traditional medicine were compounded by a lack of trust in ART and the health system generally. Accounts were also provided of people using traditional therapies and ART in combination.

*Woman: ‘[The healer] starts to cut me by a razor and think that I will be healed meanwhile that razor is adding to my illness’. (Group A)*

### Stigma and disclosure

The process revealed pronounced stigma as a cause and consequence of HIV/AIDS. Instances of children growing up unaware of their status and transmitting the disease unknowingly were recounted, as was a lack of knowledge about non-sexual routes of transmission. There were some reports of people fully accepting positive status, and being happy to disclose it. Generally, participants noted that positive status is not accepted by friends and family.

*Woman: ‘People will think that I was a prostitute, they judge’. (Group B)*

## Health systems factors

### Delays in treatment

Delays in receiving ART were reported at multiple stages. With reference again to stigma, lack of acceptance of a positive status was reported to lead to delays in seeking treatment. Participants also reported not being given ART until the condition was advanced, which, in turn, encouraged the use of traditional medicine. A lack of transport was also described as affecting people’s abilities to reach clinics, especially among groups such as the elderly.

*Woman: ‘…she will not get treatment because her CD4 count is still fine, obviously they will run to traditional herbs’. (Group C)*

### Problematic staff–patient relationships

Staff–patient relationships were identified as deeply problematic, with pronounced negativity and on occasion anger recounted towards staff. Poor staff–patient relationships were repeatedly cited as reasons for people not going for testing or adhering to treatment. Accounts of neglect of patients by nurses were also provided, with staff perceived to give preferential treatment to relatives. There were also perceived repercussions if negative comments were submitted to the clinic suggestion boxes.

*Man: ‘A lady came highly expecting and she needed treatment…the nurse looked at her and said ‘just go back home’. That lady’s husband went to the clinic and wanted to kill that nurse’. (Group A)*

*Woman: ‘They put a suggestion box so we can submit our concerns, but nothing is changing…when they take it from the box, they burn it’. (Group B)*

### Blame towards staff

Significant blame was placed on staff, especially nurses, for poor quality of care. Although there was acknowledgement of staff shortages and constraints beyond the control of nurses, it was maintained that nurses were generally at fault. Some felt that parents, and the wider community, should acknowledge the wider factors and forces that have a role in shaping the conduct of nurses. Comparatively, less blame was expressed towards doctors.

*Man: ‘The ambulance collected the lady…The nurse, instead of staying in the back with the patient, she stayed with the driver…maybe she was having a relationship with the driver. And then that lady in the back fell out of the car’. (Group A)*

### Lack of confidentiality

Lack of confidentiality was a repeated and persistent theme in discussions that reinforced the blame and negativity expressed towards clinic health workers. Specific accounts of nurses using hand signals to disclose individuals’ HIV status were made, although this was denied by others. Lack of confidentiality was also reported by the use of a colour-coded card system that revealed the HIV status of patients in clinics. These practices were reported to erode trust towards staff and actively deter people from seeking care and treatment.

*Woman: ‘…you hear them say ‘those who have blue cards go to that door’. And we now know who is living with HIV’. (Group C)*

### Lack of trust

Lack of trust was a broader issue. The government was not seen to be doing enough, especially about the health of the poor. Beliefs were expressed that the government could, if it wanted to, find a cure for HIV, and some participants blamed white people for introducing it. Corruption was believed to be rife, and this was deemed to be responsible for a large proportion of poverty. There was little hope of improvement, and a lack of confidence in politics and politicians was reported as an influence in people not voting, highlighting community disengagement more generally.

*Woman: ‘… they want to finish people, they say they are unable to cure [HIV]’. (Group C)*

*Woman: ‘… it will never be solved… that Jacob Zuma always come to [village]… He always comes and he doesn’t help us with anything’. (Group C)*

## Priorities for action

### Youth facilities

Participants described that lack of spaces and activities for young people drove them towards alcohol. Providing more spaces and positive alternatives, such as youth centres, was suggested as a means to reduce the consumption of alcohol and negative consequences.

*Man: ‘Youth are able and committed to do good things, but they don’t have a place’. (Group A)*

### Improved employment opportunities

Employment was noted as an important way to address alcohol misuse by reducing the free time of individuals. Better employment opportunities, particularly for women, were also identified as a solution to reduce financial pressures that push women into exploitative relationships, thereby addressing gender inequalities. A male participant described the view that women in particular could benefit from greater employment opportunities.

*Woman: ‘… help us get jobs, especially us who don’t have husbands, before we get involved in relationships… if I can find a job, I will not suffer’. (Group C)*

*Man: ‘…if there are jobs*…*it has to go to women so that many things can be reduced’. (Group C)*

### Government intervention/expanding community-based engagement and health promotion

Several participants expressed a desire for expanded community based health promotion to educate people about health, and to improve information on and awareness of people who are not attending or consulting at clinics. Enforcing marital regulations was also viewed as a solution to reducing extramarital affairs and HIV transmission.

*Man: ‘If they are bound by law [on marital fidelity] there will be no disease spreading’. (Group C)*

### Timely treatment and outreach

Providing health education before ART was judged to be ineffective, and it was suggested that education given simultaneously with ART would accelerate the benefits of treatment. Participants also noted the importance of extending outreach services to respond to people who were too scared or unable to attend the clinics, and to provide care and support for those who may not be served by the current system. Participants identified the need for peer support groups in outreach work. In particular, they felt that partaking in the study had been beneficial and educational, and they expressed desires for more education and participatory activities in the community in partnership with researchers and authorities.

*Woman: ‘If we could [provide treatment] at their homes they won’t be scared and the high rate of deaths would decrease’. (Group B)*

*Man: ‘People need to be taught how [HIV] works, not just in a workshop of a few people like us’. (Group A)*

## Discussion

### Local knowledge on HIV-related mortality

Blame, stigma and mistrust were woven through the narratives, characterising the causes and contributors of HIV-related mortality identified. Nurses were often viewed as negligent, fraudulent or otherwise to blame for poor quality care. This was paradoxical given the appreciation of broader contextual and systems constraints on the quality of services. Systemic constraints on nurses’ morale and performance have been documented elsewhere in South Africa, with nurses reporting depersonalisation, high occupational demands and limited organisational support.^62^ Doctors received relatively more sympathetic attitudes for challenging working conditions. Doctors are, however, rarely located in clinics. Patients have more contact time with nurses than doctors, which may make them a more accessible target for blame. A further explanation is gendered. Nursing is generally considered a female occupation, and women are traditionally expected to be caring.

The concept of blaming ‘others’ is important in public health education. People may not perceive health education/promotion messages as relevant because of implicit avoidance of issues that are threatening. ‘Othering’ also provides a route to avoid responsibility.^63^ Again, gender perspectives can help explain the finding that teenage girls and single mothers were blamed for HIV transmission, mainly by other women, several of whom in similar age groups and circumstances. Niehaus and Jonsson have argued that men externalise personal responsibility more than women.^64^ According to this view, women may accept greater personal responsibility but may project this onto other women, particularly of lower social status. The research supports this assertion.

Pronounced stigma was also conveyed towards PLHIV. It is well established in South Africa and elsewhere that families shun HIV-positive relatives or hide their status from the community. This discourages appropriate care and support, further feeding stigma and hindering treatment.^65^ Stigma is also internalised: shame associated with HIV/AIDS was frequently described by participants reporting on personal experience and perceptions. The dual nature of stigma is consistent with research demonstrating significant levels of externalised stigma and discrimination, with even greater levels of internalised stigma among PLHIV in South Africa.^66^ Our findings support earlier work noting breaches of confidentiality surrounding HIV status in this area,^52^ which may serve to further exacerbate the stigmatisation of PLHIV.

Widespread blame and stigma around HIV-related mortality contribute to a pervasive lack of trust within and outside the health system. The issue of trust is particularly important in view of the broader health systems approach.^46^ According to this perspective, the health system can be seen as a core social institution,^47^ where structure and functions reflect and reinforce community values and norms of how people interact and navigate the system.^48^ Choices, and trust, in health systems enable individuals to exercise control, acting as agents instead of victims in influencing their own health and life situations. It is noteworthy that a distinct absence of choice, autonomy and control over the causes and contributors of HIV-related mortality was a common feature of the discussions.

A pervasive lack of trust requires solutions within and beyond the health system. It is relevant in this sense that changing social norms through community involvement is viewed as essential for controlling HIV/AIDS.^67^ According to a people-centred health systems perspective, the effectiveness of the health system correlates with the quality of the human relationships involved.^68^ Previous research has found that staff felt that certain patient groups, for example, teenage mothers, use services inappropriately and undeservingly.^69^ Consequently, staff may treat these groups unsympathetically, worsening staff–patient relationships and arguably further excluding those already marginalised. This could help explain the lack of trust and poor staff–patient relationships documented here.

### A practice theory interpretation

Social theory can provide further explanations of the findings. Building on models of health systems as core social institutions, practice theory was adopted as a frame to consider the findings.^70^ Practice theory extends the classic sociological dualism of *structure* and *agency*, asserting that social practices, rather than individually acting agents or macrosocial structures, are the central site of social ordering. Practice-based analyses explore how routine and habitual ways of knowing and doing, which constitute people’s everyday lives, powerfully shape health and well-being, including the norms around issues such as eligibility for healthcare, blame, stigma and mistrust.

In this context, the problematic patient–staff relationships cannot be interpreted in terms of individual failures and prejudices (of doctors, nurses or patients), or even as simple results of the broader failures and shortages in the health system. Instead, they are seen as part, as well as a consequence, of different and sometimes conflicting practices, which involve both individuals and institutions. It is argued in this paper that blame was often unfairly directed towards nurses. Approached through the practice theory lens, blame can be seen as depending on the integration of three main elements defined in practice theory: *materials*, for example, differently coloured health cards, available and timely ART and health services infrastructures; *competence*, for example, understandings of HIV prevention, causes and treatments, profound demotivation, demoralisation and burnout; and *meaning*, for example, gendered expectations of care in nursing. Understood as a social practice, the blaming of nurses interacts with, and is sustained by, other practices, including denial of HIV status, relationship building with clinic staff, participating in support groups or enacting gendered norms of masculinity and femininity.

The practice-based approach highlights the interconnectedness of different processes and relationships affecting HIV/AIDS, both within and outside the health system. It suggests that the issues in the community, as well as their possible solutions, cannot be considered in isolation but rather in relation to various practices, which involve individual staff and patients, wider institutions and systems and the multiple interactions between them. This shifts the focus of interventions away from individuals as autonomous and rational agents towards practices as coproduced, interactional and relational.

Extending this line of argument into policy and planning, programmes to reduce stigma through widening community participation could help foster trust through greater accountability and improved relationships. Accounting for local causality through education and participation are solutions with the potential to break the cycle of blame, stigma and mistrust. Understanding how people account for causality, and consequently place blame and accountability, could improve interactions between health workers and communities, reshape public health messages and reduce blame. Practice theory contributes to this understanding.

### Methodological reflections

PAR subscribes to a relativist paradigm, which seeks to disrupt conventional analytical distinctions between subjects and objects and transfer power towards those most directly affected during the process. PAR is therefore a political process concerned with power and knowledge as transformative for action.^45^ The approach is distinct from, and complementary to, clinical and epidemiological perspectives.

From a relativist perspective, rigour is achieved by conceptualising and reconceptualising contextualised interpretations and reflexivity. Validity is considered with reference to plausibility, relevance and authenticity.^71–73^ In health policy and systems research (HPSR), analytical generalisability, that is, seeking to generate concepts and themes with relevance in different settings, is important. Through the repeated interactions, time devoted to developing a deliberative process and the multilevel analysis and interpretation of data, we met rigour criteria appropriate for our design. It should be noted that statistical generalisability is not a concern as relativist enquiry does not seek to attain this.

Considering appropriate rigour criteria for PAR, participants were recruited to prioritise partnerships and prior engagements. During data collection, individual views and experiences were elicited and systematised into collective forms of knowledge through a process that built relationships, engagement, participant control and trust. The renditions of causes and contributors of HIV-related mortality were also rigorously verified by the discussion groups, and the findings are consistent with studies on HIV in similar areas documenting blame, stigmatisation and power asymmetries in relationships.^16 21 62–66^

We developed community knowledge on how HIV is understood, experienced, treated and acted on in this setting, and we identified priorities for action. Within the time and resources available, it was possible to initiate the process, develop collective assessments and appraise actions. Data and priority actions were subsequently fed back to the provincial directorate,^74^ and a joint feedback forum was held with Department of Health representatives and participants from all discussion groups.^75 76^ In these engagements, the combination of quantitative (VA) and qualitative evidence was welcomed as providing powerful, veracious and granular representations of the communities’ experiences of HIV.

Although we maintained and extended links in communities, engaged with communities and health systems stakeholders to develop research questions, interpret data and write up findings, because  of the time and resources available, we did not engage in a process of acting on the evidence developed. Without progressing into action, and learning from action, PAR was not fully achieved.^45^ Given the active and instrumental approach to knowledge coproduction, the process can only be described as an initialisation of PAR in this setting.

Through the activities undertaken, however, willingness and commitment have been secured to continue the process into a collective reflection and action cycle embedded in routine health systems functions.^77^ This will add a crucial link to inform and understand how change occurs in health systems, by which means, for whom and the role of evidence in the process. Lessons gained from the work presented here will be incorporated. These include careful and continual attention to, and reflection on, who participates, how and through which means, the spaces, structure and support for the process, as well as communication, inclusion and evaluation of outcomes. Approaches to expand participation related to this work, and generally, are described in more detail elsewhere. 53 78

The results suggest that PAR in the context of HDSS has an important role in providing robust evidence on health systems functioning. As stated by Sheikh and colleagues in a recent series on HPSR, ‘Health systems are seen as…technological solutions rather than grounded in social and political realities. The local political cultures and practices are how policies get implemented’.^79^ It follows that participatory research adopting a focus on implementation, health inequalities and coproduction can help to understand how health is shaped in health systems. The next steps are to extend the roles of participants in the process, include the perspectives most disadvantaged and engage in the health system at different levels to move towards an ongoing process of action and learning from action.

## Conclusions

HIV/AIDS is a multifaceted issue. Governments need to work with related agencies and organisations to address a range of social and health systems factors. Policy interventions that focus on routine and habitual social processes and practices may provide a route through which to address HIV-related mortality.

A practice theory interpretation offers insights into views of health systems as a core social institutions, suggesting that the causes and contributory factors identified relating to HIV/AIDS are interdependent and constituted through various practices. Viewed through this lens, the health system itself can be understood as a complex arrangement of practices, involving different materials, competences and meanings. With social practices, rather than individual behaviours, responsible for shaping patterns of health and well-being, interventions need to focus on understanding and intervening into specific practices. These complex and intersecting practices, when considered as a locus for policy interventions, may provide routes to develop more positive and productive relationships and effective care.

Research on service organisation and delivery that involves communities may help to address the pervasive blame, mistrust and stigma that characterise HIV-related mortality in this setting. PAR methods provide robust evidence that, when conducted as part of a broader HPSR approach, can translate into policy, action and learning from action to help break the cycle of marginalisation and disengagement and to improve trust within and outside the health system. When conducted in an ongoing partnership with HDSS and health authorities, the process has the potential to foster inclusion and build trust in health systems through greater accountability and improved staff–patient relationships.

## Footnotes

i VA is a surveillance method used in over 45 low and middle-income countries to ascertain probable medical causes of death among people who die outside health facilities and/or without complete registration. VA consists of two main stages. First, a questionnaire interview is conducted by trained fieldworkers with final caregivers of the deceased (usually close family members) based on validated and standardised instruments to collect information on medical signs and symptoms of the deceased, and on the circumstances of death. Second, the interview data are interpreted to determine probable medical cause(s) of death. When undertaken at scale, for example, in health and demographic surveillance, disease burdens can be ascertained in otherwise undocumented populations.^80 81^
